# User-reported quality of care: findings from the first round of the People's Voice Survey in 14 countries

**DOI:** 10.1016/S2214-109X(23)00495-3

**Published:** 2023-12-11

**Authors:** Todd P Lewis, Munir Kassa, Neena R Kapoor, Catherine Arsenault, Rodrigo Bazua-Lobato, Rashmi Dayalu, Günther Fink, Theodros Getachew, Prashant Jarhyan, Hwa-Young Lee, Agustina Mazzoni, Jesus Medina-Ranilla, Inbarani Naidoo, Ashenif Tadele, Margaret E Kruk

**Affiliations:** a Department of Global Health and Population, Harvard T H Chan School of Public Health, Boston, MA, USA; b Minister's Office, Ministry of Health, Addis Ababa, Ethiopia; c Department of Epidemiology and Public Health, Swiss Tropical and Public Health Institute, Allschwil, Switzerland; d University of Basel, Basel, Switzerland; e Health System & Reproductive Health Research Directorate, Ethiopian Public Health Institute, Addis Ababa, Ethiopia; f Public Health Foundation of India, Gurugram, India; g Graduate School of Public Health and Healthcare Management, The Catholic University of Korea, Seoul, South Korea; h Institute for Clinical Effectiveness and Health Policy, Buenos Aires, Argentina; i School of Public Health, Cayetano Heredia University, Lima, Peru; j Centre for Community Based Research, Public Health, Societies & Belonging, Human Sciences Research Council, Durban, South Africa

## Abstract

High-quality care is essential for improving health outcomes, although many health systems struggle to maintain good quality. We use data from the People's Voice Survey—a nationally representative survey conducted in 14 high-income, middle-income, and low-income countries—to describe user-reported quality of most recent health care in the past 12 months. We described ratings for 14 measures of care competence, system competence, and user experience and assessed the relationship between visit quality factors and user recommendation of the facility. We disaggregated the data by high-need and underserved groups. The proportion of respondents rating their most recent visit as high quality ranged from 25% in Laos to 74% in the USA. The mean facility recommendation score was 7·7 out of 10. Individuals with high needs or who are underserved reported lower-quality services on average across countries. Countries with high health expenditure per capita tended to have better care ratings than countries with low health expenditure. Visit quality factors explained a high proportion of variation in facility recommendations relative to facility or demographic factors. These results show that user-reported quality is low but increases with high national health expenditure. Elevating care quality will require monitoring and improvements on multiple dimensions of care quality, especially in public systems.

This is the second in a **Series** of six papers about the People's Voice Survey on health system performance. All papers in the Series are available at www.thelancet.com/series/peoples-voice-survey

## Background

High-quality processes of health care are crucial to improving health outcomes and building confidence in the health system. In 2018, the *Lancet Global Health* Commission on high-quality health systems in the Sustainable Development Goals era identified three domains of care processes: competent care, competent systems, and user experience.^1^ Competent care refers to evidence-based health-care visits and includes systematic patient assessments, accurate diagnoses, provision of appropriate treatments, and proper patient counselling. A competent system is one in which the entire system functions well for the patient beyond the content of a single visit and includes important components of quality, such as safety, disease prevention and detection, service continuity and integration, and timely care. Positive user experience, including respect, absence of discrimination and abuse, and good communication, is both intrinsically valuable to patients and instrumental in improving retention in care and adherence to treatments. When done optimally, these processes of care work together to provide patient-centred care that improves health outcomes.

Both in literature on health systems and in practice, there has been growing recognition of the importance of the patient's perspective in assessing the quality of health care.^2^ An increasing number of indicators and instruments are available to help evaluate quality of care and monitor the improvement of services.^3^, ^4^ In some cases, patient-reported quality indicators have been incorporated into routine health system assessment and provider payment systems.^5^, ^6^ These factors increasingly play a central role in service delivery reforms.

Despite the importance of the user's perspective to providing high-quality care, many health system decision makers do not adequately consider user-reported quality when implementing or evaluating health policies. Existing surveys often measure a small subset of the full range of care processes. Measurement is often restricted to quality for specific populations (eg, women aged 15–49 years in Demographic and Health Surveys) or to assessment of clinical observations and settings (eg, patient exit interviews done through service provision assessments).^7^, ^8^ There is a need for a targeted set of cross-nationally comparable patient-reported measures to better understand and improve quality of service delivery.

**Key messages**

• Content of care and experience of the health system is unsatisfactory on average, with marked variation across the 14 countries in this analysis
• Countries with a high health expenditure per capita tended to have better ratings of care than countries with a low health expenditure, although greater investment does not guarantee higher quality
• Underserved groups, users with the highest health needs, and those who used public primary care facilities tended to rate quality worse
• In all countries, users considered multiple aspects of quality, including both technical and interpersonal quality, when rating their most recent health facility visit; provider skills and respect from staff had the strongest influence on user willingness to recommend the health facility
• In contrast to common measures of patient satisfaction, facility recommendation score was largely explained by aspects of health-care visit quality as opposed to demographic, health system, or broader societal factors; facility recommendation might be a more useful indicator than traditional measures of patient satisfaction for assessing care quality from the user perspective
• Governments seeking to raise perceptions of quality, which is crucial for improving health outcomes, must regularly measure care quality from the population perspective and improve on multiple dimensions of care competence and user experience; improving quality is especially crucial for the sickest and most vulnerable population groups

This analysis builds on work from the high-quality health systems Commission using the People's Voice Survey (PVS)—a new instrument that measures user-reported quality among adults in any setting. The PVS includes a broader range of quality measures than previous surveys and enables comparison across different regions, health systems, and national health expenditures; such comparisons can be useful for benchmarking quality and identifying novel strategies to strengthen quality of care. The PVS is a product of the Quality Evidence for Health Systems Transformation Network—an initiative focused on measuring and improving health system quality through multicountry partnerships; the PVS is the first new tool to emerge from this network. This analysis is the first to use data on user-reported quality of a recent health-care visit from the PVS.

In this Series paper, we used data from the PVS done in 14 high-income, middle-income, and low-income countries to better understand quality at the point of care and in health systems. We described the quality of the most recent visit and broader system competence from all care in the past 12 months from the patient's perspective. We disaggregated overall quality ratings by high-need and underserved groups and facility type to understand how quality is distributed among policy-relevant populations. Finally, we assessed the relationship between a respondent's recommendation of the facility and elements of visit quality to understand the components of quality that matter most to people and to test the utility of facility recommendation as a measure of overall quality performance.

## Study sample

This analysis used PVS data from the following 14 countries, collected from May 9, 2022, to April 3, 2023: Ethiopia, Kenya, South Africa, Peru, Colombia, Mexico, Uruguay, Argentina (the province of Mendoza only), Laos, India, South Korea, Italy, the UK, and the USA. A nationally representative random sample of 1000–2000 adults aged 18 years and older per country were primarily selected via random-digit dialling, although known-list sampling or online probability panels were used when necessary to reach the population (sampling method by country is available in appendix 1). Respondents were interviewed about their health, health-care use, ratings of most recent health-care visit, and related topics. Interviews were done via telephone or web-based survey and supplemented with in-person interviewing in areas with less than 80% mobile phone ownership, such as in rural regions of Kenya and Ethiopia. Our sample includes all respondents who reported an in-person visit to a health facility in the 12 months preceding the survey with complete data on variables of interest. Participants who had home or telemedicine visits only in the past 12 months were excluded from analysis.

The Harvard University Institutional Review Board deemed this research exempt from full review, and additional local ethics approval was obtained as required in relevant countries.

## Outcome definition

We included two summary outcomes of facility-based care in the past 12 months: overall quality of the most recent visit and recommendation of the facility of the most recent visit. The most recent visit has been used elsewhere as a proxy for recent care and helps to maximise recall.^9^ The rating of overall quality of the most recent visit was binary and was defined as whether the respondent rated the overall quality of their most recent visit in the past 12 months as excellent or very good (ie, 1) versus good, fair, or poor (ie, 0) on a five-point Likert scale. To measure facility recommendation, we asked participants the following survey question: “Using a scale from zero to 10, where zero means you definitely would not recommend and 10 means you definitely would recommend, how likely is it that you would recommend this healthcare provider or facility to a friend or family member?” This item, a measure of potential future recommendation, has been used extensively in customer satisfaction surveys and has been proposed as a more intuitive indicator for evaluating health services.^10^ The resulting recommendation score is the number between zero and ten selected by the respondent. Full survey questions and response options are available in appendix 2 (pp 1–2).

## Covariates

We constructed a conceptual framework to identify processes of care and population factors that might affect facility recommendation (appendix 2 p 3). The resulting framework included 12 components of care competence, system competence, and user experience and 12 population factors. Care competence measures included provider knowledge and skills, clarity of provider explanations, and equipment and supplies. User experience measures included visit duration, visit waiting time, appointment waiting time (not asked in all countries), courtesy of facility staff, respect from the provider, and involvement in care decisions. Indicators were binary, with a 1 indicating the component of quality was excellent or very good and a 0 indicating good, fair, or poor. All measures were assessed for the 12 months before the survey interview date.

We included three measures of system competence that refer to all care in the past 12 months rather than at the most recent visit. System competence measures included three binary items: whether a mistake was perceived in care or treatment or not, whether the respondent experienced unfair treatment or discrimination or not, and whether there was an unmet need for care (ie, foregone care in the past 12 months despite needing medical attention) or not. A 1 indicated the item was avoided (eg, no mistake was reported) and a 0 indicated it was not (eg, a mistake was reported).

Individuals with high health needs were those with low self-rated health and low self-rated mental health, defined as those who rated their health as fair or poor (ie, 1) versus excellent, very good, or good (ie, 0). High-need older adults were defined as those 50 years and older who reported a chronic illness. Women of reproductive age were defined as female respondents aged 18–49 years.

Underserved groups included those on low incomes, those with low educational attainment, and those with low activation. Low income was defined as approximately the bottom tertile of total monthly (or annual) household or individual income in each country. Low educational attainment was defined as those who had no formal education or completed only primary or secondary school. Low activation was defined as those who reported they were not confident they could bring up concerns to their provider or they were not confident they were responsible for managing their own health.

Private versus public health facility ownership and primary versus secondary health facility were defined by country (appendix 2 pp 4–6). Facilities managed by social security systems were grouped with public health facilities for analysis. Reason for visit was defined as an urgent or new health problem, follow-up care for a chronic condition, or a routine visit. Health insurance coverage was excluded for countries with nominal universal national health insurance systems. Health expenditure per capita was measured as the most recent estimate available expressed in international dollars at purchasing power parity from the WHO Global Health Expenditure database.

## Analysis

We first described the characteristics of the sample in each country by demographic group, health need group, and facility and visit type. We assessed quality through descriptive analyses of the two primary outcomes of quality and the 12 components of care competence, user experience, and system competence by country and in the sample overall. To understand how quality is distributed across key groups in the population, we calculated the percentage of respondents who rated overall quality of the most recent visit highly in stratified analyses among each high-need and underserved group, by facility type, and by national health expenditure per capita.

We constructed three multivariable linear regression models for the association between facility recommendation and each component of quality among low, medium, and high health expenditure countries, as we expected the importance of quality to vary by health system strength. All models controlled for high-need group status, underserved group status, facility and reason for visit, and country fixed effects to account for unmeasured health system and other relevant national-level confounders. We calculated the variance in facility recommendation score explained by covariates and the proportion of R-squared attributable to components of visit quality. System competence measures were not included in regression models as they did not pertain to the facility of most recent visit. Health insurance status was excluded from our models as this variable was not applicable in all countries. We clustered SEs to account for the sampling approach for in-person interviews in Ethiopia and Kenya. All descriptive analyses were weighted to represent the national adult population. Analyses were done with Stata version 16.1.

## Overall quality ratings

The PVS surveyed a total of 23 230 adults across the 14 countries. Of these participants, 15 294 respondents had at least one in-person visit at a health facility in the past 12 months. Country-specific samples ranged from 618 in Italy to 1887 in South Korea (table 1). Across countries, the majority of respondents were younger than 50 years, except in South Korea, Italy, and the UK. The proportion of the population with low self-rated health ranged from 16·0% in the USA to 46·5% in Peru. More than 50% of visits were at public facilities in all countries except India, South Korea, and the USA.

**Table 1:** Characteristics of respondents reporting at least one health-care visit in the past 12 months

		**Ethiopia (n=1528)**	**Kenya (n=1398)**	**South Africa (n=1444)**	**Peru (n=890)**	**Colombia (n=946)**	**Mexico (n=639)**	**Uruguay (n=976)**	**Argentina (n=918)**	**Laos (n=1018)**	**India (n=653)**	**South Korea (n=1887)**	**Italy (n=618)**	**UK (n=1084)**	**USA (n=1296)**
**Demographics**															
Sex															
	Female	777 (51%)	771 (55%)	821 (57%)	463 (52%)	509 (54%)	329 (51%)	546 (56%)	578 (63%)	550 (54%)	311 (48%)	964 (51%)	314 (51%)	560 (52%)	685 (53%)
	Male	751 (49%)	627 (45%)	623 (43%)	427 (48%)	437 (46%)	310 (49%)	430 (44%)	340 (37%)	468 (46%)	342 (52%)	923 (49%)	304 (49%)	524 (48%)	611 (47%)
Older than 50 years		284 (19%)	258 (18%)	379 (26%)	280 (31%)	322 (34%)	185 (29%)	402 (41%)	401 (44%)	277 (27%)	123 (19%)	965 (51%)	341 (55%)	553 (51%)	641 (49%)
Education to secondary school or lower		1383 (91%)	1240 (89%)	1291 (89%)	735 (83%)	725 (77%)	504 (79%)	857 (88%)	615 (67%)	854 (84%)	395 (60%)	536 (28%)	512 (83%)	249 (23%)	486 (38%)
Low income		616 (40%)	992 (71%)	563 (39%)	437 (49%)	297 (31%)	346 (54%)	439 (45%)	391 (43%)	279 (27%)	349 (53%)	557 (30%)	273 (44%)	326 (30%)	415 (32%)
Rural residence		1036 (68%)	974 (70%)	437 (30%)	145 (16%)	133 (14%)	121 (19%)	65 (7%)	60 (7%)	707 (69%)	326 (50%)	249 (13%)	27 (4%)	103 (10%)	167 (13%)
Low activation		730 (48%)	623 (45%)	627 (43%)	601 (68%)	509 (54%)	357 (56%)	418 (43%)	404 (44%)	456 (45%)	433 (66%)	1716 (91%)	494 (80%)	678 (63%)	631 (49%)
**Health needs**															
Low self-rated health		558 (37%)	290 (21%)	503 (35%)	414 (47%)	252 (27%)	253 (40%)	234 (24%)	228 (25%)	307 (30%)	288 (44%)	784 (42%)	194 (31%)	283 (26%)	208 (16%)
Low self-rated mental health		350 (23%)	128 (9%)	305 (21%)	242 (27%)	139 (15%)	140 (22%)	154 (16%)	125 (14%)	236 (23%)	222 (34%)	462 (24%)	80 (13%)	260 (24%)	207 (16%)
Chronic illness		306 (20%)	282 (20%)	485 (34%)	249 (28%)	267 (28%)	163 (26%)	443 (45%)	400 (44%)	307 (30%)	119 (18%)	767 (41%)	230 (37%)	590 (54%)	557 (43%)
High-need older adults		122 (8%)	92 (7%)	243 (17%)	143 (16%)	169 (18%)	108 (17%)	273 (28%)	250 (27%)	139 (14%)	42 (6%)	482 (26%)	171 (28%)	356 (33%)	337 (26%)
Women of reproductive age		666 (44%)	634 (43%)	571 (40%)	318 (36%)	327 (35%)	223 (34%)	314 (32%)	328 (36%)	416 (41%)	281 (43%)	445 (24%)	141 (23%)	288 (27%)	408 (31%)
**Characteristics of most recent visit**															
Private facility		308 (20%)	418 (30%)	352 (24%)	279 (31%)	443 (47%)	189 (30%)	123 (13%)	340 (37%)	215 (21%)	399 (61%)	1384 (73%)	239 (39%)	62 (6%)	1218 (94%)
Secondary facility		143 (9%)	596 (43%)	346 (24%)	357 (40%)	526 (56%)	168 (26%)	629 (64%)	425 (46%)	631 (62%)	332 (51%)	947 (50%)	340 (55%)	187 (17%)	94 (7%)
Reason for visit															
	New problem	991 (65%)	970 (69%)	384 (27%)	220 (25%)	217 (23%)	304 (48%)	245 (25%)	287 (31%)	416 (41%)	394 (60%)	482 (26%)	151 (24%)	412 (38%)	308 (24%)
	Follow-up care	257 (17%)	163 (12%)	438 (30%)	155 (17%)	236 (25%)	131 (21%)	301 (31%)	195 (21%)	90 (9%)	155 (24%)	767 (41%)	138 (22%)	411 (38%)	384 (30%)
	Preventive care	282 (18%)	266 (19%)	624 (43%)	516 (58%)	495 (52%)	205 (32%)	432 (44%)	438 (48%)	513 (50%)	105 (16%)	639 (34%)	330 (53%)	263 (24%)	605 (47%)
Uninsured		574 (38%)	953 (68%)	..	106 (12%)	..	101 (16%)	..	..	..	453 (69%)	14 (1%)	..	..	71 (5%)
**Health expenditure**															
Health expenditure per capita, purchasing power parity (INT$)		75	208	1187	712	1204	1111	2310	2199	212	211	3521	3998	5087	10921

Argentina includes the province of Mendoza only. Low income is defined as those in approximately the bottom tertile of income in each country's sample. Low activation was defined as those who reported they were not confident they could bring up concerns to their provider or they were not confident they were responsible for managing their own health. Low self-rated health and low self-rated mental health are defined as people who rate their own health or mental health as fair or poor. High-need older adults are adults older than 50 years who reported a chronic illness. Women of reproductive age are defined as women aged 18–49 years. Private *vs* public facilities in Colombia are defined by whether the respondent had public or private insurance coverage. Insurance status is not available in countries with universal insurance schemes. Health expenditure per capita is measured in international dollars. Frequencies are rounded up to the nearest whole number as the data are weighted to each national population, which can produce decimal frequencies.

**Figure 1:**
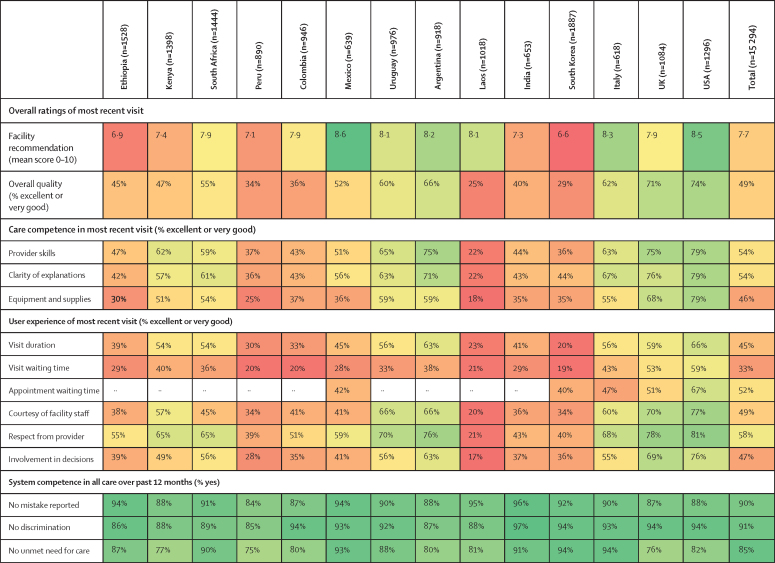
Ratings of most recent health-care visit and system competence in the past 12 months Red indicates worse performance and green indicates better performance. Argentina includes the province of Mendoza only. Appointment waiting time is missing for countries where this question was not included in the survey.

Recommendation score for the facility of most recent visit was a mean of 7·7 out of 10 across all countries (figure 1). The lowest mean recommendation score was 6·6 in South Korea and the highest was 8·6 in Mexico. The percentage of respondents rating overall quality of the most recent visit as excellent or very good was 49% in the entire sample, ranging from 25% in Laos and 29% in South Korea to 71% in the UK and 74% in the USA.

Across all of the countries, ratings were lowest for visit waiting time (33% in the full sample, ranging from 19% in South Korea to 59% in the USA) and visit duration (45% in the full sample, ranging from 20% in South Korea to 66% in the USA). When considering all quality components, respondents in Laos and South Korea tended to rate quality lowest, whereas respondents in the USA rated their care most highly.

Summative system competence measures, including whether the respondent perceived mistakes in care or treatment, unfair treatment or discrimination, and unmet need for care, were at 75% or above in all countries (figure 1). Summative system competence measures were lowest in Peru, with 84% of respondents reporting no mistake made, 85% reporting no discrimination, and 75% of respondents reporting no unmet need for care.

## Quality ratings by health status and underserved group

**Figure 2:**
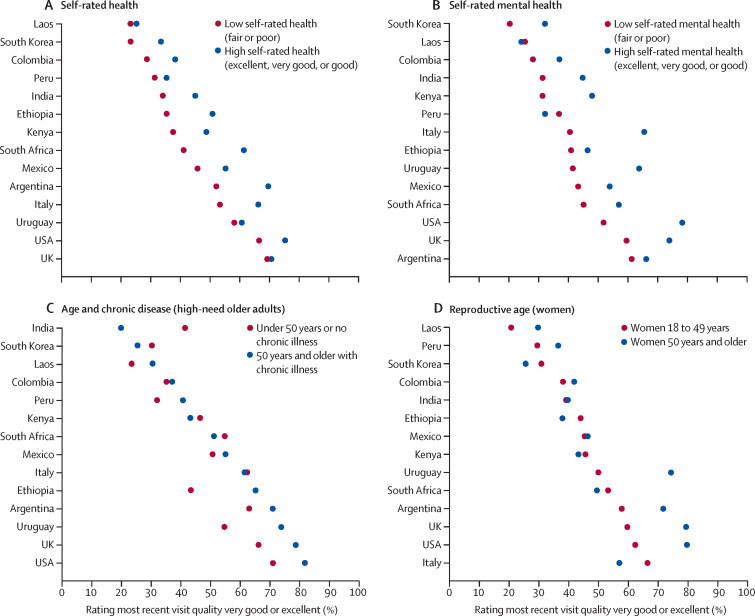
Proportion of respondents rating quality of most recent health-care visit as very good or excellent, by health need Low self-rated health or mental health=fair or poor. High self-rated health or mental health=excellent, very good, or good.

In stratified analyses, adults with low self-rated health and low self-rated mental health rated overall quality of the most recent visit lower than those with high self-rated health and high self-rated mental health in all countries, except in Laos and Peru where differences were small (figure 2; appendix 2 pp 7–8). Differences in ratings of most recent health-care visit quality between people who have high needs and are older and people who are younger or healthier were highly heterogeneous across countries. Ratings were also mixed among women of reproductive age, who reported worse quality at most recent health-care visit than older women in nine countries, with pronounced differences in all high-income countries except South Korea.

**Figure 3:**
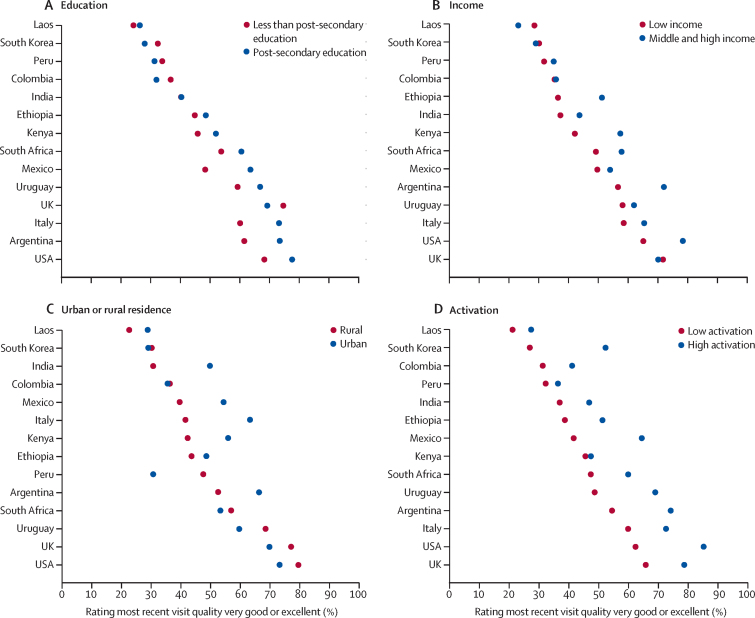
Proportion of respondents rating quality of most recent health-care visit as very good or excellent, by underserved groups

In countries with high ratings of overall quality of most recent health-care visit, respondents who had completed post-secondary education and those with high income tended to rate the recent health-care visit more highly than respondents with lower levels of education and people with a low income (figure 3; appendix 2 pp 7–8). In countries with low quality ratings, the inverse tended to be true: people with low income or lower levels of education tended to rate the most recent health-care visit more highly than people with high income or who had completed post-secondary education. However, differences were smaller than in countries with high quality ratings. The difference in care ratings between urban and rural populations was highly heterogeneous by country. In Argentina, India, Italy, Kenya, and Mexico, respondents living in urban areas rated the most recent health-care visit more highly than respondents living in rural areas, and differences between subgroups were larger than in other countries. In every country, respondents with high activation rated the most recent health-care visit more highly than respondents with low activation.

## Quality ratings by facility type and national health spending

**Figure 4:**
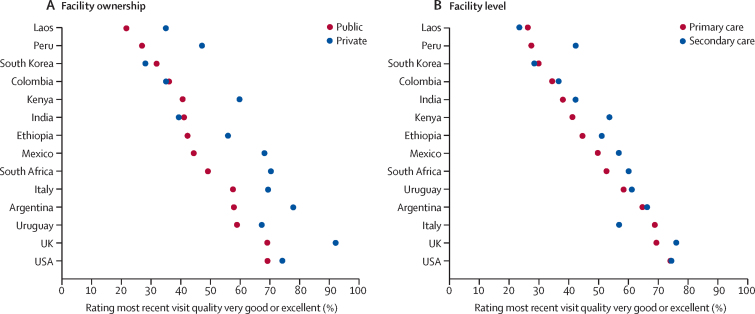
Proportion of respondents rating quality of most recent health-care visit as very good or excellent, by facility type

In most countries, most recent health-care visit at private facilities was rated more highly than at public facilities or differences were small (figure 4; appendix 2 pp 7–8). Similarly, the percentage of people rating the most recent health-care visit as very good or excellent was higher in secondary care facilities than in primary care facilities in all but three countries.

**Figure 5:**
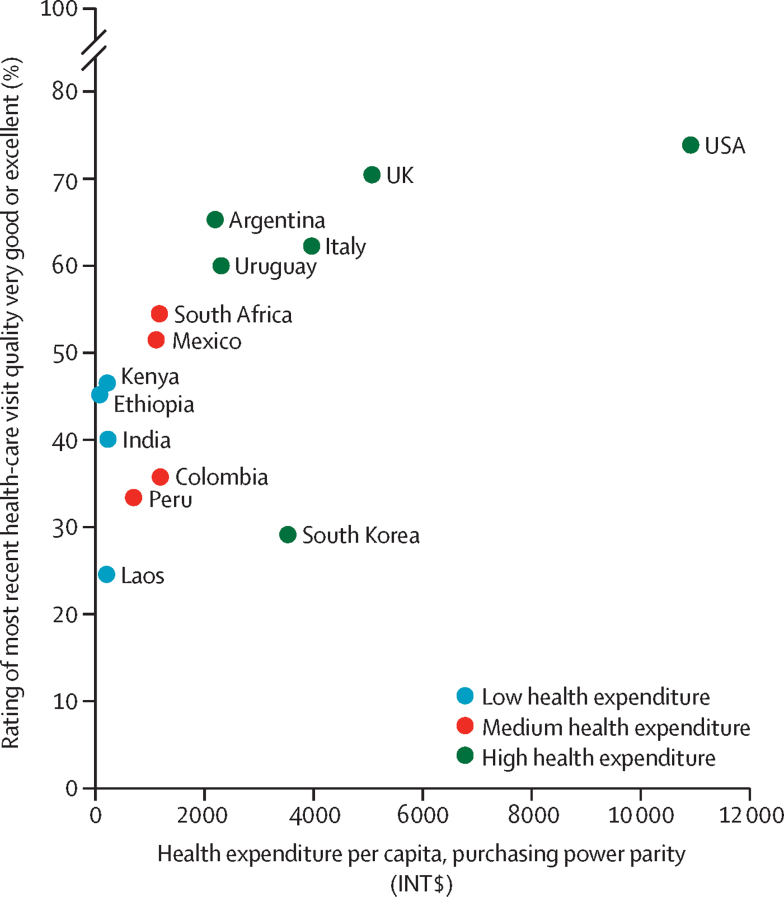
Percentage ratings of overall quality of most recent health-care visit by national health expenditure per capita (international $)

Ratings of overall quality of the most recent health-care visit trended strongly with health expenditure per capita in most countries, with respondents rating care higher in countries with high health expenditure than in countries with low health expenditure (figure 5). The exception was South Korea, which had a health expenditure per capita of INT$3521 but the second lowest proportion of respondents rating care highly at 29%.

## Associations between facility recommendation and visit quality, facility, and demographic factors

In the regression models, all care competence and user experience indicators were significantly associated with facility recommendation, except clarity of provider explanations and visit waiting time in low-health-expenditure countries (table 2). In those countries, the coefficients for provider skills, availability of equipment and supplies, and courtesy of facility staff were largest in magnitude. In medium-expenditure and high-expenditure countries, the coefficients for respect from the provider and the courtesy of facility staff were largest in magnitude among user experience factors. Among care competence factors, clarity of explanations was largest for middle-expenditure countries and provider skills was largest for high-expenditure countries. Women, older adults, and people using private facilities had significantly higher recommendation scores than men, younger adults, and people using public facilities in all models. Across health expenditure groups, people with low activation had significantly lower recommendation scores than people with high activation. R^2^, which measures the amount of variation in the outcome explained by covariates on a scale from 0 to 1, was 0·12 in low-expenditure countries, 0·29 in middle-expenditure countries, and 0·48 in high-expenditure countries. Visit quality factors alone accounted for 58% of observed variation in low-expenditure countries, 86% in middle-expenditure countries, and 96% in high-expenditure countries in each model.

**Table 2:** Associations between facility recommendation and visit quality, facility, and demographic factors

		**Low health expenditure (n=4680)**		**Medium health expenditure (n=3893)**		**High health expenditure (n=6721)**	
		β (95% CI)	p value	β (95% CI)	p value	β (95% CI)	p value
**Visit quality factors (ie, excellent or very good *vs* good, fair, or poor)**							
Provider skills		0·36 (0·20 to 0·53)	<0·0001	0·37 (0·18 to 0·55)	0·0001	0·60 (0·45 to 0·74)	<0·0001
Clarity of explanations		0·14 (−0·03 to 0·32)	0·10	0·45 (0·27 to 0·63)	<0·0001	0·49 (0·35 to 0·63)	<0·0001
Equipment and supplies		0·33 (0·16 to 0·50)	0·0002	0·39 (0·23 to 0·55)	<0·0001	0·39 (0·27 to 0·51)	<0·0001
Visit duration		0·18 (0·02 to 0·35)	0·033	0·36 (0·19 to 0·53)	<0·0001	0·39 (0·28 to 0·51)	<0·0001
Visit waiting time		0·13 (−0·04 to 0·29)	0·12	0·21 (0·06 to 0·36)	0·0068	0·29 (0·20 to 0·38)	<0·0001
Courtesy of facility staff		0·31 (0·15 to 0·47)	0·0002	0·67 (0·51 to 0·83)	<0·0001	0·54 (0·42 to 0·67)	<0·0001
Respect from provider		0·24 (0·08 to 0·41)	0·0047	0·50 (0·31 to 0·68)	<0·0001	0·60 (0·46 to 0·74)	<0·0001
Involvement in care decisions		0·25 (0·08 to 0·42)	0·0037	0·18 (0·00 to 0·36)	0·044	0·40 (0·28 to 0·53)	<0·0001
**Facility and visit type**							
Private facility		0·28 (0·14 to 0·43)	0·0002	0·53 (0·39 to 0·67)	<0·0001	0·15 (0·05 to 0·24)	0·0032
Secondary care facility		0·04 (−0·10 to 0·19)	0·56	0·20 (0·06 to 0·34)	0·0051	−0·02 (−0·11 to 0·08)	0·74
Reason for visit (reference: urgent care)							
	Follow-up care	−0·12 (−0·33 to 0·09)	0·27	0·08 (−0·13 to 0·28)	0·47	0·13 (0·01 to 0·24)	0·028
	Preventive care	0·02 (−0·13 to 0·16)	0·80	0·10 (−0·06 to 0·25)	0·21	0·21 (0·11 to 0·31)	<0·0001
**Demographic factors**							
Female		0·21 (0·08 to 0·34)	0·0014	0·22 (0·08 to 0·35)	0·0016	0·10 (0·02 to 0·18)	0·012
Age 50 years and older		0·30 (0·13 to 0·46)	0·0054	0·26 (0·09 to 0·43)	0·0022	0·36 (0·27 to 0·44)	<0·0001
Secondary school education or lower		0·30 (0·16 to 0·43)	<0·0001	0·27 (0·13 to 0·41)	0·0002	0·05 (−0·04 to 0·14)	0·30
Low income		−0·04 (−0·20 to 0·13)	0·67	0·23 (0·07 to 0·39)	0·0047	−0·04 (−0·13 to 0·05)	0·40
Rural residence		−0·32 (−0·47 to −0·18)	<0·0001	−0·13 (−0·34 to 0·09)	0·25	0·05 (−0·08 to 0·18)	0·48
Low activation		−0·21 (−0·34 to −0·07)	0·0024	−0·25 (−0·38 to −0·11)	0·0004	−0·23 (−0·32 to −0·14)	<0·0001
Low self-rated health		−0·15 (−0·31 to 0·02)	0·079	−0·15 (−0·32 to 0·01)	0·073	0·09 (−0·01 to 0·20)	0·069
Chronic illness		−0·16 (−0·34 to 0·01)	0·067	−0·04 (−0·22 to 0·14)	0·67	−0·01 (−0·10 to 0·08)	0·88
**Country**							
Reference: Ethiopia							
	Kenya	0·05 (−0·14 to 0·24)	0·59	..	..	..	..
	Laos	1·06 (0·88 to 1·24)	<0·0001	..	..	..	..
	India	−0·02 (−0·30 to 0·26)	0·91	..	..	..	..
Reference: Peru							
	South Africa	..	..	0·02 (−0·18 to 0·22)	0·88	..	..
	Mexico	..	..	0·97 (0·75 to 1·19)	<0·0001	..	..
	Colombia	..	..	0·37 (0·18 to 0·57)	0·0002	..	..
Reference: South Korea							
	Uruguay	..	..	..	..	0·40 (0·23 to 0·57)	<0·0001
	Argentina	..	..	..	..	0·35 (0·19 to 0·52)	<0·0001
	Italy	..	..	..	..	0·59 (0·44 to 0·74)	<0·0001
	UK	..	..	..	..	0·02 (−0·14 to 0·18)	0·78
	USA	..	..	..	..	0·17 (0·03 to 0·31)	0·017
Variance explained (R^2^)		0·12	..	0·29	..	0·48	..

The outcome is a continuous variable measuring how likely the respondent would be to recommend their most recently used health-care facility to friends and family on a scale from 0 to 10. Visit quality factors, while measured with a Likert scale, were made binary with a 1 indicating the component of quality was excellent or very good and a 0 indicating good, fair, or poor. Low activation was defined as those who reported they were not confident they could bring up concerns to their provider or they were not confident they were responsible for managing their own health. Low self-rated health and low self-rated mental health are defined as those who rate their health or mental health as fair or poor. Private facilities *vs* public facilities in Colombia are defined by whether the respondent had public or private insurance coverage. Argentina includes the province of Mendoza only. Coefficients are the mean change in facility recommendation score associated with a one-unit change in each predictor.

## Discussion

In this analysis, we used novel, prospectively collected and population-representative data from 14 high-income, middle-income, and low-income countries to investigate quality of care. We found that ratings of overall quality, care competence, and user experience of the most recent visit were low on average, with substantial heterogeneity across countries. We also found that populations most in need of good health care tended to rate quality worse than their healthier counterparts. Provider knowledge and skills, courtesy from facility staff, and respect shown by providers were among the strongest predictors of facility recommendation across countries. Finally, we found that facility recommendation is highly reflective of visit quality ratings and might better capture user-reported quality than patient satisfaction measures.

Our results show that overall quality is low in these 14 countries. This finding reflects entrenched poor quality among health systems globally and underinvestment in the aspects of quality that matter most to the public. Previous studies in low-income and middle-income countries (LMICs) showed that providers performed fewer than half of evidence-based care actions and approximately a third of patients reported disrespectful care, short consultations, poor communication, or long waiting times.^1^, ^11^, ^12^ High-income-country health systems have their own challenges, such as financial pressures, high demand for care, and cuts to social care.^13^, ^14^

System competence measures, assessed as experience of a given item in all care over the previous 12 months, were uniformly higher across countries than were care competence or user experience measures. However, these results still signal a need for attention. In Peru, 16% of respondents reported experiencing discrimination or perceiving a mistake in their care in the past 12 months, which is notable given that health system contacts are typically infrequent and short in duration.

Our findings show that quality ratings tended to increase according to national-level health expenditure. These results bolster previous findings that health and social service expenditures are positively associated with health outcomes and show that a similar relationship holds for quality at the point of care. Greater investment in the health system can improve quality through multiple channels, such as improved pre-service education for providers, better governance for quality, improved organisation of service delivery, and reduced disparities.^15^, ^16^ However, this relationship did not always hold. South Korea, a country with the fourth-highest health expenditure in our sample, was among the lowest for ratings of care competence and user experience. This finding and other identified quality differentials between countries could in part reflect higher expectations for care in some countries compared with others.^17^ Furthermore, many health systems in countries at all income levels have strained to meet the demands of the COVID-19 pandemic, which has probably influenced these quality ratings differentially by setting.^18^

We found that user-reported quality was lower among people with high health needs than in people with low health needs in many countries. Those with low self-rated health and low self-rated mental health reported lower care quality than people with high self-rated health and mental health consistently across countries. There might be a direct relationship in which poor health produces a negative effect that patients attach to health-care providers and facilities, leading to low satisfaction with health services—a relationship that is found elsewhere.^19^, ^20^, ^21^ There was more heterogeneity among women of reproductive age and adults who are older and have high needs. For example, in most high-health-expenditure countries, adults who are older than 50 years and have high needs rated quality higher than people who are younger or healthier, and women of reproductive age rated quality lower than women who are older. Observed rates of satisfaction among adults who are older than 50 years and have high needs in our analysis were consistent with those of the 2017 Commonwealth Fund International Health Policy Survey, an 11-country survey that included the USA and UK, which found high rates of dissatisfaction with quality of care in the past year among adults older than 65 years with high needs.^22^ Advancing age tends to have a positive association with patient satisfaction.^23^ Patients with chronic illness, which is more common among older adults, typically rate their care more highly than other users.^24^ Patients who are older might also have lower expectations of service quality given their familiarity with the health system relative to younger patients.^25^

We also found that quality of the most recent visit was worse for low education, low income, low activation, and rural populations, with heterogeneity across countries. Previous multicountry research has shown an inequitable distribution of quality: poor and rural populations often receive worse quality care in high-income and low-income countries alike.^1^, ^9^, ^26^ We also found that education had an inverse relationship with facility recommendation, but income did not. This finding suggests that high-income individuals can identify or access high-quality facilities, and more educated individuals might have higher baseline expectations and thus be more selective. Finally, we found that patients with high activation reported better quality of care in every country than people with low activation, which might be due to their ability to identify better providers or extract better quality of care during a visit.^27^ Strategies to build demand for quality is an area ready for further research.^28^

For all countries in our sample, except India and South Korea, our results indicated that people perceived quality to be higher in private systems than public systems. This finding aligns with results from other multicountry surveys that found private facilities outperformed public and social security facilities on measures of user experience.^29^ However, differences in quality between the public and private sectors vary by context. A systematic review of LMICs found that private providers were less likely to follow practice standards, were less efficient, and had poorer patient outcomes than providers in the public system.^30^

In seven of 14 countries, we found that a small proportion of respondents rated quality at secondary care facilities higher than primary care facilities, although this finding was significant for middle-income countries only in adjusted analyses. This finding might reflect disproportionate investment in secondary care facilities in middle-income countries and population perception that more advanced facilities provide higher quality care.^31^

In our regression models, nearly every component of visit quality was significantly associated with facility recommendation. In all countries, respect shown by the provider and courtesy of the facility staff were prominent. These results are consistent with a previous analysis of 41 countries that found that dignity (including respect and privacy) was the second most important domain of interpersonal quality after prompt attention.^32^ In high-expenditure and low-expenditure countries, the coefficient for provider knowledge and skills was large in magnitude, suggesting that users consider technical quality of care when assessing their health facility.

Across our models, a high proportion of observed variance was explained by elements of visit quality. By contrast, demographic factors, facility and visit type, and country fixed effects provided little additional explanatory power. These results run counter to those found by Bleich and colleagues,^33^ who analysed patient satisfaction data from 12 European countries and found that satisfaction depends more on societal factors external to the health system than on experience of care and concluded that patient satisfaction measures might be of restricted use in assessing care quality. Thus, patient satisfaction measures, although useful for capturing the role of broader social, political, and contextual factors, might be poorly suited to assessing care quality. Our findings suggest that the facility recommendation item in the PVS better reflects visit quality and might be a more useful measure of overall quality performance than previously used patient satisfaction measures. In addition to its relationship to visit quality, facility recommendation score might be less cognitively demanding for respondents and produces a continuous response that requires fewer assumptions in analysis than other measures, such as Likert scale responses.^10^ Facility recommendation score is an important complement to patient satisfaction indicators currently in use.

We also found our models' explanatory power increased with increased health expenditure. For low-health-expenditure countries, there are probably components of care or other factors that meaningfully influence facility recommendation that are unmeasured in our survey. This low explanatory power could reflect reduced facility choice in LMICs, where there might be fewer accessible, high-quality facilities available.^34^ Additional research on the aspects of care that generate high ratings in LMICs is required.

Our analysis is strengthened through use of novel, nationally representative data of the adult population and by measurement of a wide-ranging, standardised set of measures of care and system competence and user experience. However, the analysis is subject to limitations. Survey data are subject to recall bias, although our survey items are measured over the past 12 months—a shorter recall period compared with some similar surveys.^7^ Our survey data ask about the most recent visit only to minimise recall bias—a common although imperfect proxy for broader health-care use. Our regression estimates possibly could be confounded by unobserved factors. Our sample was on average poorer than the national population; thus, our low-income group is larger than the true population distribution in some countries.

These results can help inform national policy decisions. First, our findings show that populations are paying attention to quality and are often dissatisfied with their care. Second, the results show that users consider multiple distinct measures of quality when assessing their care. Measuring these components can direct policy makers to high-priority areas for investment. Third, measurement of user-reported quality might benefit from further testing of novel indicators, such as facility recommendation, which better reflect the quality of a health-care visit. Fourth, policy makers should pay close attention to raising the bar on quality for all people, especially people with the highest need or from the most marginalised groups, which will be particularly important with increasing age and multimorbidity in the population. Finally, although competent care and good user experience are crucial conduits to improved health outcomes, they are also intrinsically valuable—they ensure users receive good clinical care and, at minimum, the basic dignity that they should receive from the health system.

## Data sharing

Individual-level, de-identified data from the People's Voice Survey will be publicly available in mid-2024. Data will be available on the Harvard Dataverse. The survey instrument and data dictionary will be available upon publication.

## Declaration of interests

We declare no competing interests.
