# Supplementary material for: User-reported quality of care: findings from the first round of the People's Voice Survey in 14 countries
Source: Lancet Glob Health. 2023 Dec 11;12(1):e112–22. doi: 10.1016/S2214-109X(23)00495-3 (PMC10716624; doi:10.1016/S2214-109X(23)00495-3)
Supplement: Supplementary appendix 2 [file mmc2.pdf]

THE LANCET
Global Health

Supplementary appendix 2

This appendix formed part of the original submission and has been peer reviewed. We post it as supplied by the authors.

Supplement to: Lewis TP, Kassa M, Kapoor NR, et al. User-reported quality of care: findings from the first round of the People's Voice Survey in 14 countries. *Lancet Glob Health* 2023; published online Dec 11. https://doi.org/10.1016/S2214-109X(23)00495-3.

Supplement A: Additional methods and data

Supplement A table 1: People's Voice Survey questions on care competence, user experience and system competence

Variable	Survey question	Response options
**Ratings of the last health care visit in the past 12 months**		
	The following questions are about your experiences in your last healthcare visit for a new or ongoing health problem or a regular checkup. Please include only your last in-person visit to a trained healthcare provider. This excludes pharmacy or shops or traditional providers.<br><br>Thinking about this last visit, how would you rate the following…?	
Overall quality of last visit	the overall quality of care you received	Excellent, Very good, Good, Fair, Poor
Provider skills	the knowledge and skills of your provider	Excellent, Very good, Good, Fair, Poor
Equipment and supplies	the equipment and supplies that the provider had available, such as medical equipment or access to lab tests	Excellent, Very good, Good, Fair, Poor
Respect from provider	the level of respect your provider showed you	Excellent, Very good, Good, Fair, Poor
Clarity of explanations	Whether your provider explained things in a way you could understand	Excellent, Very good, Good, Fair, Poor
Involvement in decisions	whether your provider involved you as much as you wanted to be in decisions about your care	Excellent, Very good, Good, Fair, Poor
Visit duration	the amount of time your provider spent with you	Excellent, Very good, Good, Fair, Poor
Visit wait time	the amount of time you waited before being seen	Excellent, Very good, Good, Fair, Poor
Courtesy of facility staff	the courtesy and helpfulness of the healthcare facility staff, other than your provider	Excellent, Very good, Good, Fair, Poor
Appointment wait time	how long it took for you to get this appointment	Excellent, Very good, Good, Fair, Poor

Facility recommendation	Using a scale from 0 to 10, where 0 means you definitely would not recommend and 10 means you definitely would recommend, how likely is it that you would recommend this healthcare provider or facility to a friend or family member?	0 to 10
**Experience of system competence in all care over the past 12 months**		
	Thinking about the last 12 months, could you tell me if the following events have happened to you personally?	
No mistake made	You thought a medical mistake was made in your treatment or care.	Yes, No
No discrimination	You were treated unfairly or discriminated against by a doctor, nurse, or another healthcare provider.	Yes, No
No unmet need for care	In the last 12 months, was there a time when you had a health problem and needed medical attention, but you did not get healthcare from a provider?	Yes, No

Supplement A figure 1: High-quality processes of care: Indicators from the People's Voice Survey

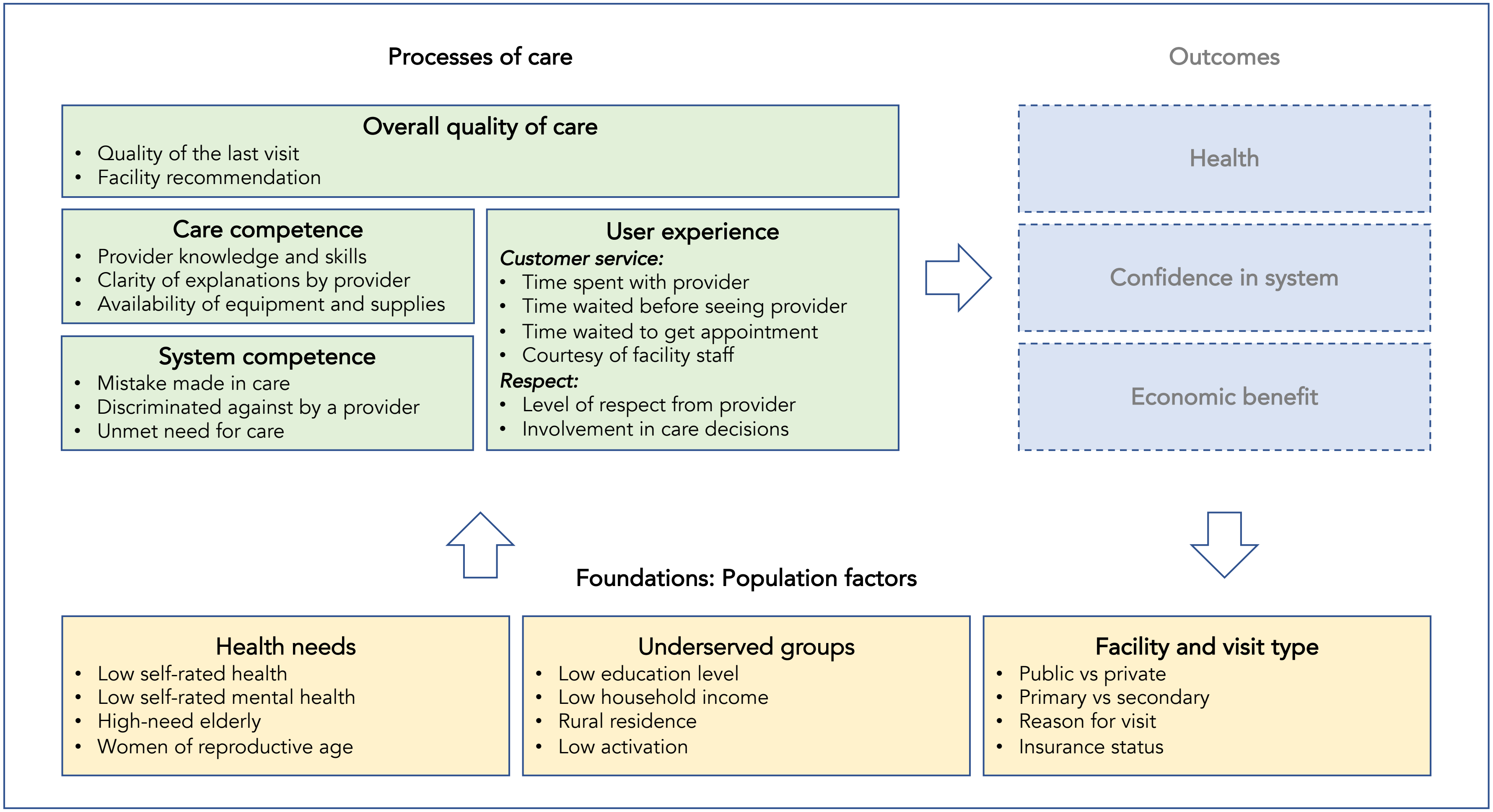

Supplement A table 2: Facility ownership type and level by country

		AR	CO	ET	GB	IN	IT	KE
**Facility ownership for last visit in the last 12 months**	**Public**	Public, OSEP, Other 'obras sociales' (Example: OSPE, OSDIPP), PAMI	Public (based on insurance type): SISBEN, subsidized, no insurance	Public	NHS facility, HSC facility	Public	Public	Public
	**Private**	Prepaid or private (Example OSDE, GALENO, OMINT, MEDIFÉ or similar ones)	Private (based on insurance type): private, contributory, contributory prepaid	Private, NGO, Faith-based	Private facility	Private (for-profit), NGO/Faith-based	Private accredited by SSN, Private not accredited by SSN	Private, NGO, Faith-based
**Facility level for last visit in the last 12 months**	**Primary**	Health Center / Policlinic / Doctor's Office, OSEP Cerca / Delegación / Doctor's Office, Other primary care facility	Health center, Other primary care facility, Health center, Other primary care	Health post, Health center, Mobile clinic, Primary Hospital, Lower clinic, Medium clinic, NGO/Faith-based health center/clinic, Higher clinic/specialty clinic	General practitioner's (GPs) office NHS health centre or NHS walk-in centre HSC health centre or HSC walk-in centre Private medical centre or medical group Urgent care centre Private clinic	Sub-centre/Health and Wellness Centre, Primary Health Centre, Community Health Centre, Informal Providers (rural medical providers (RMP)), Private clinic, Private nursing home	General practitioner's office, Outpatient clinic	Government dispensary, Government health center or clinic, Community health worker or outreach, Mobile clinic, Private clinic or doctor's office, Private medical center, NGO/faith-based clinic
	**Secondary**	Hospital, Clinic / Sanatorium / Hospital / OSEP Central, Other secondary care facility or higher	Hospital, Clinic, Another secondary care facility, Hospital, Clinic, Another secondary care facility, Other secondary care	General Hospital, Referral /specialized hospital, Hospital /specialty center, NGO/Faith-based hospital	Same day emergency care (SDEC) Hospital emergency room Hospital outpatient department (doctor's office based in hospital)	District Hospital, Medical College, Private hospital, Faith-based hospital (religion or sect-based facility)	Hospital outpatient department (doctor's office based in hospital), Hospital emergency room	Government /district /county hospital, Private hospital, NGO/faith-based hospital

LA	MX	PE	ZA	KR	GB	US	UY
Government hospital, Government health center	Secretaria de Salud o Servicios Estatales de Salud, IMSS-Bienestar (antes IMSS prospera o IMSS Oportunidades), El IMSS, El ISSSTE/ISSSTE Estatal, PEMEX, Defensa or Marina, Other	Public (based on insurance type): SIS, EsSalud, Sanidad de las Fuerzas Armadas y Policiales	Public	Public	NHS facility, HSC facility	Free community clinic or health center (e.g., Planned Parenthood or other clinics that are free of charge like Federally Qualified Health Centers), Veteran's Affairs, Military, or Indian Health Service clinic or health center	Public, mutual
Private hospital, Private clinic, NGO/Faith cllinic	Establecimiento privado	Private	Private, NGO, Faith-based	Private (non-profit organization/religion-related)	Private facility	Doctor's office, clinic, or health center, Urgent care clinic, Hospital emergency room, Hospital outpatient department (doctor's office based in hospital)	Private
Government health center, Private clinic, NGO/Faith clinic	Puesto de salud, Clinica o unidad de medicina familiar, Clínica o unidad de medicina familiar, clínica o unidad de consulta externa, módulo de medecina familiar o puesto periférico, Brigada móvil de salud, Centros de salud o centro de primer contacto, Brigada móvil de salud o unidad médica móvil, Unidad de salud o	Health post, Health center, Post/health center, Other primary care facility, Post/health center, Private office, other primary care facility	Mobile clinic, Ward-based outreach care, Health posts, Department of health clinic, Municipal clinic, Department of health community center, General practitioners practices, Private clinics, Private health centers, Faith-based or mission clinic	Health center, Clinic	General practitioner's (GPs) office<br>NHS health centre or NHS walk-in centre<br>HSC health centre or HSC walk-in centre<br>Private medical centre or medical group<br>Urgent care centre<br>Private clinic	Doctor's office, clinic, or health center, Urgent care clinic, Free community clinic or health center (e.g., Planned Parenthood or other clinics that are free of charge like Federally Qualified Health Centers), Veteran's Affairs, Military, or Indian Health Service clinic or health center	Health center, Other primary care facility, Health center, Other primary care facility, Health center, Other primary care facility, Other primary care

	Unidad de Médica Rural, Clínica o unidad de consulta externa, Consultorio anexo a farmacia, Consultorio médico privado o grupos de consultorios de especialidades de atención ambulatoria							
Government hospital, Private hospital	Hospital general, Centro Médico Nacional o Hospitales de especialidades como hospital de ginecología y obstetricia, pediatría, oncología, cardiología, oftalmología, etc., Hospital general o regional, Centro médico Nacional o Hospital de especialidades, Hospital civil, municipal, general, o regional, Hospital general, hospital rural o centro de atención rural obstétrica, Institutos Nacionales con hospitales monotemáticos (por ejemplo, Instituto Nacional de salud mental), Hospital de especialidades, Hospital general privado, Hospital de especialidades privado	Hospital, National Institute, Another secondary care facility, Clinic, Higher or specialty clinic, Other secondary care facility, Hospital/specialty centre	District hospital, Department of health hospital (regional, Tertiary and Central hospitals), Specialist private practices, Private hospital, Faith-based or mission hospital	Secondary Hospital (30-100 beds or less, regardless of the number of medical subjects), Tertiary general hospital (mostly university hospitals)	Same day emergency care (SDEC) Hospital emergency room Hospital outpatient department (doctor's office based in hospital)	Hospital emergency room, Hospital outpatient department (doctor's office based in hospital)	Hospital or sanatorium, Other secondary care facility, Clinic or sanatorium, Clinic or sanatorium, Other secondary care facility	

Notes: Argentina includes the Province of Mendoza only. "Appointment wait time" is missing for countries where this question was not included in the survey. Country codes: ET=Ethiopia, KE=Kenya, ZA=South Africa, PE=Peru, CO=Colombia, MX=Mexico, UY=Uruguay, AR=Argentina, LA=Laos, IN=India, KR=South Korea, It=Italy, GB=United Kingdom, US=United States.

Supplement A table 3: Ratings of overall quality of the last visit in the past 12 months by health need, underserved groups, and facility type

	ET	KE	ZA	PE	CO	MX	UY	AR	LA	IN	KR	IT	GB	US
Sample size =	1528	1398	1444	890	946	639	976	918	1018	653	1887	618	1084	1296
**Health need groups**														
**Self-rated health**														
High self-rated health	51.0%	48.9%	61.6%	35.4%	38.4%	55.4%	60.9%	69.9%	25.3%	45.2%	33.5%	66.5%	71.0%	75.6%
Low self-rated health	35.5%	37.7%	41.2%	31.3%	28.4%	45.9%	58.4%	52.3%	23.2%	34.2%	23.2%	53.5%	69.5%	66.8%
**Self-rated mental health**														
High self-rated mental health	46.6%	48.1%	57.0%	32.2%	37.0%	53.9%	63.8%	66.2%	24.4%	44.9%	32.1%	65.6%	74.0%	78.4%
Low self-rated mental health	41.0%	31.5%	45.1%	37.0%	28.6%	43.4%	41.5%	61.3%	25.5%	31.4%	20.5%	40.6%	59.6%	51.9%
**High-need older adults**														
Under 50 years old and/or no chronic illness	43.6%	46.8%	55.1%	32.1%	35.5%	50.9%	54.9%	63.4%	23.7%	41.7%	30.5%	62.7%	66.4%	71.4%
50 years and older with chronic illness	65.5%	43.3%	51.5%	40.9%	36.8%	55.3%	74.2%	71.3%	30.6%	20.1%	25.8%	61.8%	79.1%	82.2%
**Women of reproductive age**														
Women 50 years and older	37.6%	42.9%	49.2%	36.2%	41.5%	46.2%	74.1%	71.4%	29.4%	39.5%	25.2%	56.7%	79.2%	79.4%
Women 18 to 49 years old	43.7%	45.3%	52.9%	29.1%	37.9%	45.0%	49.5%	57.4%	20.4%	38.8%	30.5%	66.1%	59.3%	61.9%
**Underserved groups**														
**Education**														
Post-secondary	48.7%	52.1%	60.6%	31.4%	32.3%	63.6%	66.9%	73.6%	26.4%	40.3%	28.0%	73.3%	69.3%	77.7%
Secondary school or lower	45.0%	45.9%	53.8%	34.0%	36.8%	48.4%	59.4%	61.6%	24.3%	40.3%	32.4%	60.1%	74.7%	68.3%
**Household income**														
Middle and highest income	51.3%	57.5%	57.8%	35.2%	35.8%	53.9%	62.0%	72.1%	23.2%	43.7%	28.9%	65.5%	70.1%	78.4%
Lowest income	36.5%	42.1%	49.2%	31.8%	35.7%	49.7%	58.2%	56.7%	28.6%	37.4%	30.2%	58.5%	71.7%	65.2%

**Urban vs rural**														
Urban	48.7%	56.1%	53.4%	30.8%	35.8%	54.4%	59.7%	66.4%	29.0%	49.8%	29.1%	63.3%	69.9%	73.4%
Rural	43.7%	42.4%	57.0%	47.6%	35.6%	39.7%	68.7%	52.7%	22.8%	30.8%	30.3%	41.7%	77.3%	79.6%
**Activation**														
High activation	51.3%	47.4%	59.9%	36.3%	41.2%	64.4%	69.0%	74.2%	27.4%	46.8%	52.4%	72.6%	78.6%	85.3%
Low activation	38.7%	45.6%	47.4%	32.2%	31.0%	41.5%	48.6%	54.5%	21.3%	37.0%	27.0%	59.8%	65.7%	62.5%
**Facility type**														
**Facility ownership**														
Public	42.6%	40.9%	49.3%	27.2%	36.3%	44.7%	59.3%	58.1%	21.8%	41.4%	32.1%	57.9%	69.3%	69.5%
Private	55.9%	60.0%	70.5%	47.4%	35.1%	68.4%	67.5%	78.1%	35.2%	39.7%	28.2%	69.6%	92.6%	74.5%
**Facility level**														
Primary	44.7%	41.3%	52.7%	27.6%	34.5%	49.8%	58.5%	64.9%	26.4%	38.2%	30.0%	69.0%	69.4%	74.2%
Secondary	51.2%	53.7%	60.3%	42.4%	36.7%	57.0%	61.3%	66.3%	23.6%	42.4%	28.6%	57.0%	76.1%	74.4%

Notes: Argentina includes the Province of Mendoza only. Activation is defined as the respondent being very confident (versus somewhat, not too, or not at all confident) they can bring up concerns to their provider without being asked and they are responsible for managing their overall health. Public ownership includes social health insurance and social security systems in relevant countries. Private versus public facilities in Colombia are defined by whether the respondent had public or private insurance coverage. Country codes: ET=Ethiopia, KE=Kenya, ZA=South Africa, PE=Peru, CO=Colombia, MX=Mexico, UY=Uruguay, AR=Argentina, LA=Laos, IN=India, KR=South Korea, It=Italy, GB=United Kingdom, US=United States.
